# The prognostic power of major pathological response in esophageal squamous cell carcinoma patients undergoing neoadjuvant chemoimmunotherapy: a multi-center cohort study

**DOI:** 10.3389/fimmu.2025.1599526

**Published:** 2025-07-07

**Authors:** Shuhan Xie, Shijie Huang, Zilu Tang, Hai Zhang, Jinxin Xu, Sunkui Ke, Jinbiao Xie, Rongyu Xu, Ying Chen, Zhinuan Hong, Mingqiang Kang

**Affiliations:** ^1^ Department of Thoracic Surgery, Fujian Medical University Union Hospital, Fuzhou, China; ^2^ Key Laboratory of Cardio-Thoracic Surgery (Fujian Medical University), Fujian Province University, Fuzhou, China; ^3^ Key Laboratory of Ministry of Education for Gastrointestinal Cancer, Fujian Medical University, Fuzhou, China; ^4^ Fujian Key Laboratory of Tumor Microbiology, Fujian Medical University, Fuzhou, China; ^5^ Department of Cardiothoracic Surgery, The Affiliated Hospital of Putian University, Putian, China; ^6^ Department of Thoracic Surgery, Quanzhou First Hospital, Quanzhou, China; ^7^ Department of Thoracic Surgery, Gaozhou People’s Hospital, Gaozhou, Guangdong, China; ^8^ Department of Thoracic Surgery, Zhongshan Hospital of Xiamen University, School of Medicine, Xiamen University, Xiamen, China

**Keywords:** neoadjuvant chemoimmunotherapy, major pathological response, adjuvant therapy, pathological complete response, recurrence pattern

## Abstract

**Background:**

For esophageal squamous cell carcinoma(ESCC), neoadjuvant chemoimmunotherapy (nICT) constitutes an innovative therapeutic strategy. However, The relationship between its short-term efficacy and long-term prognosis requires further clarification. Therefore, this study aims to evaluate the prognostic significance of major pathological response (MPR) in ESCC patients receiving nICT.

**Method:**

This is a retrospective multi-center study enrolling 306 ESCC patients undergoing nICT. The primary endpoints were recurrence-free survival (RFS) and recurrence patterns. Propensity score matching (PSM) was applied to address heterogeneity between groups. Kaplan-Meier curves and Cox regression analysis were utilized to analyze survival difference.

**Results:**

144 achieved a MPR, while 68 achieved a pathological complete response (pCR). Cox regression analysis identified MPR as an independent prognostic factor [HR = 0.48, 95%CI= (0.28 - 0.82), P = 0.007]. Survival analysis demonstrated that MPR patients experienced significantly improved RFS compared to non-MPR patients, before (P<0.001) and after PSM (P = 0.016). Importantly, the RFS of MPR patients was comparable to that of pCR patients (P = 0.319 in the unmatched cohort; P = 0.456 in the matched cohort). Furthermore, adjuvant therapy did not provide additional recurrence-free benefits for MPR patients. Compared to pCR patients, MPR patients exhibited a similar recurrence rate, with similar recurrence sites.

**Conclusion:**

MPR represents a significant prognostic indicator in ESCC patients undergoing nICT, demonstrating prognostic outcomes comparable to those of pCR. These findings indicated that MPR could function as a surrogate endpoint for pCR, potentially influencing treatment strategies by refining follow-up protocol and the implementation of adjuvant therapy.

## 1 Introduction

Esophageal cancer (EC) is the seventh most prevalent malignant neoplasm worldwide and is the sixth leading cause of cancer-related mortality (1). A significant proportion of patients with esophageal squamous cell carcinoma (ESCC) are initially diagnosed at a locally advanced stage. To improve prognosis, the standard treatment approach for these patients entails the administration of neoadjuvant therapy followed by surgical intervention.

Extensive clinical evidence supported the recommendation of neoadjuvant chemotherapy (nCT) and neoadjuvant chemoradiotherapy (nCRT) as standard treatments for locally advanced esophageal squamous cell carcinoma (LA-ESCC) patients (2, 3). With recent advancements in immunotherapy, its potential as a therapeutic strategy for LA-ESCC has garnered significant attention. The safety profile of neoadjuvant chemoimmunotherapy(nICT) is supported by evidence from multiple clinical trials (4–6), which demonstrate no significant increase in postoperative complications, such as anastomotic leakage. Furthermore, its efficacy is reinforced by elevated rates of major pathological response (MPR) and pathological complete response (pCR), both of which are critical indicators of improved treatment outcomes. In 2012, the U.S. Food and Drug Administration (FDA) recognized pCR as a surrogate endpoint for survival in studies of neoadjuvant therapy, based on the premise that pCR may serve as a predictor of clinical benefit (7). However, apart from pCR, MPR can also serve as an indicator of the sensitivity and efficacy of treatment modalities. Our prior study has demonstrated that MPR can function as a survival surrogate, providing a clinical reference and an evidence-based foundation for decision-making in ESCC patients undergoing nCT/nICT (8).

Previous research has demonstrated that EC patients treated with nCRT who exhibited microscopic residual disease faced an elevated risk of recurrence, however their overall survival paralleled that of patients achieving a pCR (9). Nevertheless, different treatment modalities have been observed to exhibit differing short-term efficacy and long-term prognoses in LA-ESCC patients (10, 11). To the best of our knowledge, there is a paucity of studies directly comparing the prognostic difference of MPR with pCR in ESCC patients undergoing nICT, underscoring the necessity for further research in this area. Therefore, this study aims to examine the prognostic value and clinical significance of MPR in ESCC patients undergoing nICT.

## 2 Method

### 2.1 Study population

This study recruited patients diagnosed with EC from five medical centers between January 1, 2019, and August 31, 2024. The inclusion criteria were (1): histologically confirmed ESCC and diagnosed as cT_3-4a_N_any_M_0_ or cT_1-2_N_+_M_0_ (2); receipt of at least one cycle of neoadjuvant chemoimmunotherapy (3); receipt of esophagectomy following neoadjuvant chemoimmunotherapy; and (4) availability of complete clinical data. Exclusion criteria included (1): the presence of other primary malignant tumors (2); unsuitability for surgical resection; and (3) undergoing salvage or palliative surgery.

### 2.2 Treatment protocol

Among the five medical centers, there are slight difference in preoperative examinations and nICT protocols; however, a high degree of consistency is maintained across all centers. Pre-treatment diagnosis and clinical staging were conducted through gastroscopy, contrast-enhanced computed tomography, and neck color Doppler ultrasound. Positron emission tomography was performed when required.

Commonly, the chemotherapy regimen consisted primarily of platinum combined with paclitaxel or docetaxel, administered once every 3 weeks. On the basis of nCT, the immune drug was applied in nICT group, using one of the following five types: camrelizumab, pembrolizumab, sintilimab, tislelizumab, and toripalimab. The treatment regimen and its dosage will be established through multidisciplinary consultations and adjusted based on the patient’s physical condition, particularly in response to adverse reactions. The efficacy of the treatment should be assessed 3–4 weeks after the completion of the final neoadjuvant therapy cycle. If surgical intervention is deemed appropriate, patients will undergo a minimally invasive esophagectomy. In instances where preoperative evaluation indicates the possibility of cervical lymph node metastasis, a three-field lymphadenectomy will be conducted.

### 2.3 Relevant definition

The primary endpoint of this study is recurrence-free survival (RFS), which is defined as the interval from the date of surgical resection to the occurrence of locoregional recurrence or/and distant metastasis. Pathological complete response (pCR) is characterized by the absence of residual tumor cells in both the primary tumor tissue and the lymph nodes. Major pathological response (MPR) refers to the presence of 10% or fewer viable tumor cells in resected tumor (12, 13). Various scoring systems were employed to evaluate the pathological response in patients with LA-ESCC undergoing neoadjuvant therapy (14). In this study, the tumor regression grade (TRG) was utilized to assess the pathological response. In accordance with the guidelines established by the College of American Pathologists (CAP) and the National Comprehensive Cancer Network (NCCN), MPR was equated to TRG 0-1, while it corresponds to TRG 1–2 under the Mandard scoring system (15–17).

### 2.4 Follow-up and adjuvant therapy

The postoperative follow-up schedule for ESCC patients was typically consistent in our study. During the first two years, ESCC patients underwent routine follow-up evaluations every 3 to 6 months. From the third to the fifth year, follow-ups were performed every 6 months, and subsequently on an annual basis. Furthermore, follow-up was carried out via outpatient visits and telephone consultations in our study.

In our study, adjuvant therapy is advised for ESCC patients diagnosed with ypT_0-4a_N_+_M_0_. Conversely, for patients with ypT_0-4a_N_0_M_0_, especially those achieving a pCR, both surveillance and adjuvant therapy are deemed suitable options. Additionally, a multidisciplinary team conducted consultations to evaluate the need for adjuvant therapy, taking into account the postoperative pathological outcomes, the overall health status of the patients, and their personal treatment preferences. The adjuvant therapy regimens comprised chemotherapy (aCT), immunotherapy (aIT), or a combination of both therapies (aICT). The detail information of drugs used in nICT and AT was shown in **Supplementary Table 1**.

### 2.5 Statistical analysis

**Figure 1:**
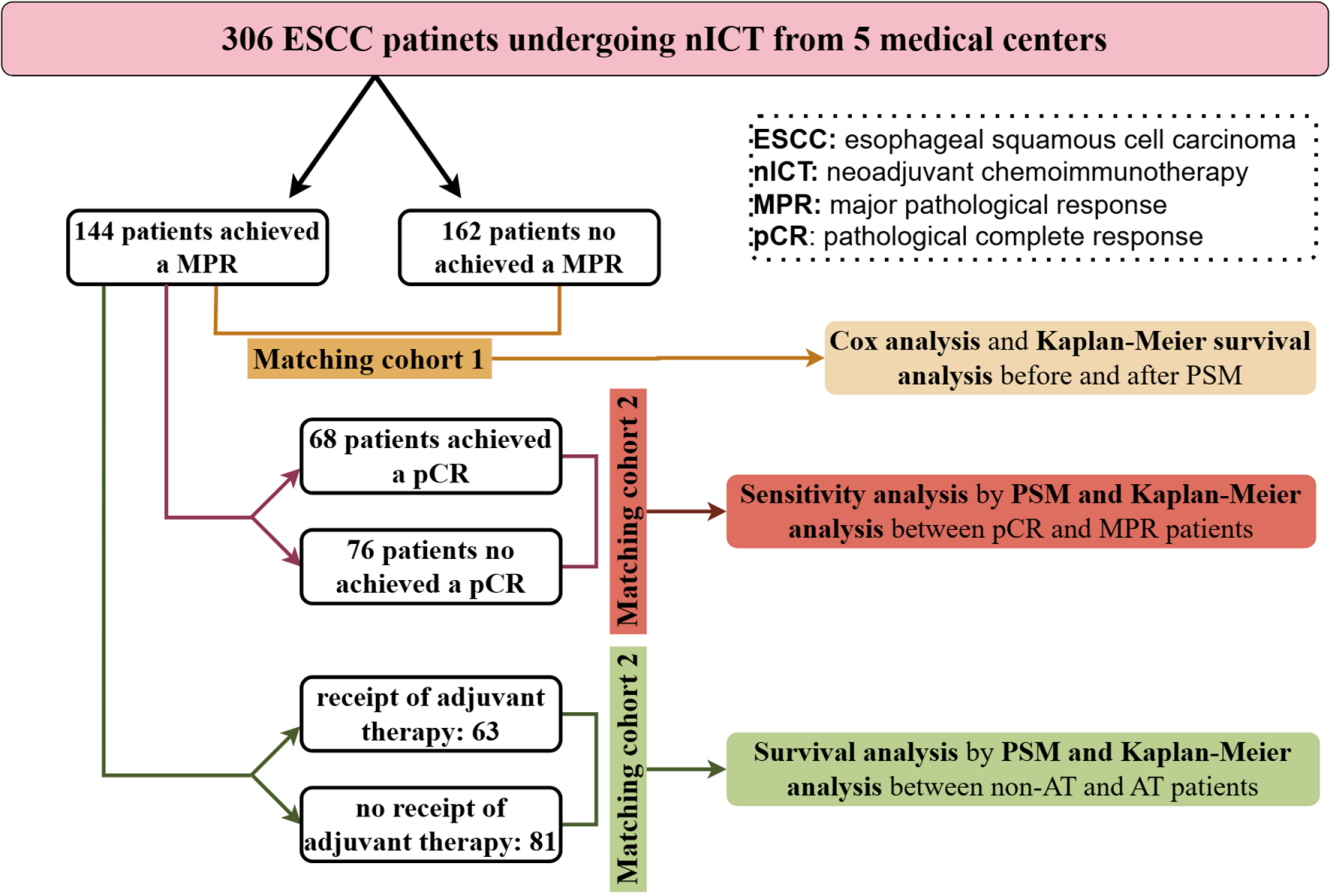
The flow chart of statistical analysis process of this study.

Categorical data were presented as counts and percentages, compared using Chi-square or Fisher's exact tests. Propensity score matching (PSM) was employed to mitigate bias arising from confounding variables, utilizing logistic regression to generate scores and implementing nearest neighbor matching without replacement, with a caliper of 0.05 and a ratio of 1:1. Matching parameters included sex, age, smoking history, drinking history, BMI, tumor location, clinical stage. Survival difference was evaluated using Kaplan-Meier survival curves. To investigate risk factors, Cox regression analysis was conducted. Variables exhibiting statistical significance in the univariate Cox regression analysis were incorporated as predictors in the multivariate analysis. Additionally, a backward stepwise regression approach was employed in the multivariate analysis. The data were processed with SPSS version 27 and R version 4.3.1. Statistical significance was set at P < 0.05. The flow chart of statistical analysis process of this study was as shown in **Figure 1**.

## 3 Results

### 3.1 Patients characteristic

In this study, a cohort of 306 patients diagnosed with ESCC and treated with nICT from five medical centers was analyzed. Among these patients, 233 cases (76.14%) were male, and 213 (69.61%) were aged 65 years or younger. Postoperative pathological outcomes showed that 144 patients achieved a MPR, while 68 attained a pCR. Following nICT and surgical intervention, 153 patients received adjuvant therapy. Detailed baseline characteristics of this patient population are presented in **Table 1**.

**Table 1:** Baseline characteristics of ESCC patients receiving nICT.

Variables	N	(%)
Sex		
Male	233	76.14%
Female	73	23.86%
Age		
≤65	213	69.61%
>65	93	30.39%
BMI		
<18.5	38	12.42%
18.5-23.9	217	70.92%
≥24	51	16.67%
Smoking history		
No	120	39.22%
Yes	186	60.78%
Drinking history		
No	182	59.48%
Yes	124	40.52%
Tumor location		
Upper	26	8.50%
Middle	171	55.88%
Lower	109	35.62%
Clinical stage		
II	75	24.51%
III	195	63.73%
Iva	36	11.76%
Neoadjuvant therapy cycle		
≤3	279	91.18%
>3	27	8.82%
ypT stage		
T_0-2_	142	46.41%
T_3-4_	164	53.59%
ypN stage		
N_0_	172	56.21%
N_1-3_	134	43.79%
pCR		
No	238	77.78%
Yes	68	22.22%
MPR		
No	162	52.94%
Yes	144	47.06%
Adjuvant therapy		
No	153	50.00%
Yes	153	50.00%

pCR, pathological complete response; MPR, major pathological response.

### 3.2 The Cox regression analysis for ESCC patients undergoing nICT

Cox regression analysis was employed to examine the independent risk factors in ESCC patients following nICT. The univariate Cox analysis identified ypT stage, ypN stage, MPR, and pCR as prognostic factors influencing this patient cohort. In the subsequent multivariate Cox analysis, ypN stage [HR= 1.88, 95%CI=(1.13 - 3.10), P = 0.014] and MPR [HR = 0.48, 95%CI= (0.28 - 0.82), P = 0.007] emerged as independent risk factors, as detailed in **Table 2**.

**Table 2:** Univariate and Multivariate Cox analysis for recurrence-free survival in ESCC patients after nICT.

Variables	Univariate Cox Analysis		Multivariate Cox Analysis	
	HR (95%CI)	P value	HR (95%CI)	P value
Sex		0.682		
male	Reference			
female	0.89 (0.50 -1.57)			
Age		0.938		
≤65	Reference			
>65	1.02 (0.61 -1.72)			
BMI		0.066		
<18.5	Reference			
18.5-23.9	0.66 (0.33 -1.31)	0.233		
≥24	1.26 (0.57 -2.78)	0.563		
Smoking history		0.796		
no	Reference			
yes	0.94 (0.58 -1.53)			
Drinking history		0.569		
no	Reference			
yes	0.87 (0.53 -1.42)			
Tumor location		0.213		
upper	Reference			
middle	0.55 (0.26 -1.15)	0.112		
lower	0.75 (0.35 -1.61)	0.465		
Clinical stage		0.114		
II	Reference			
III	1.85 (0.98 -3.48)	0.057		
IVa	1.16 (0.46 -2.94)	0.758		
ypT stage		<0.001		
T_0-2_	Reference			
T_3-4_	2.49 (1.46 -4.23)			
ypN stage		0.001		0.014
N_0_	Reference		Reference	
N_1-3_	2.23 (1.37 -3.65)		1.88 (1.13 -3.10)	
MPR		<0.001		0.007
no	Reference		Reference	
yes	0.41 (0.24 -0.69)		0.48 (0.28 -0.82)	
pCR		0.006		
no	Reference			
yes	0.31 (0.13 -0.71)			
Adjuvant therapy		0.238		
no	Reference			
yes	1.34 (0.83-2.17)			

pCR, pathological complete response; MPR, major pathological response.

### 3.3 The survival comparison of MPR patients with non-MPR patients and pCR patients

**Figure 2:**
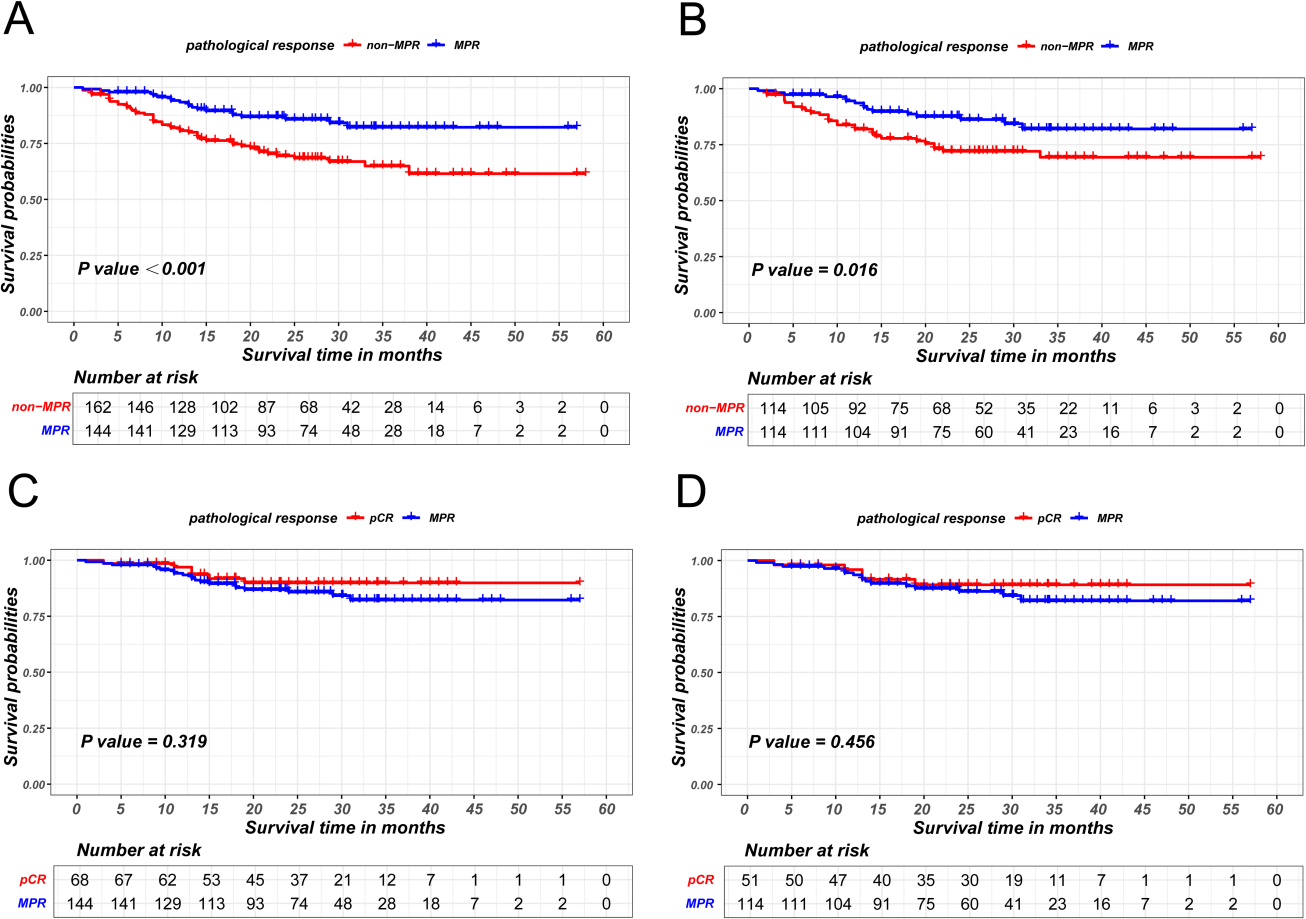
Kaplan-Meier survival curves of recurrence-free survival between non-MPR and MPR patients before PSM **(A)** and after PSM **(B)**. Kaplan-Meier survival curves of recurrence-free survival between pCR and MPR patients before PSM **(C)** and after PSM **(D)**.

The propensity score matching method was employed to mitigate baseline differences between the MPR patient group and the non-MPR patient group, as illustrated in **Table 3**. The survival curve by Kaplan-Meier analysis indicated that the recurrence-free survival of MPR patients was significantly superior than that of non-MPR patients, both before (P<0.001) and after PSM (P = 0.016), as is shown in **Figures 2A, B**.

**Table 3:** Characteristics comparison of MPR and non-MPR patients in nICT group before and after matching.

Variables	Before PSM			After PSM		
	non-MPR	MPR	P value	non-MPR	MPR	P value
Sex			0.212			0.874
male	128 (79.01%)	105 (72.92%)		89 (78.07%)	88 (77.19%)	
female	34 (20.99%)	39 (27.08%)		25 (21.93%)	26 (22.81%)	
Age			0.040			0.770
≤65	121 (74.69%)	92 (63.89%)		80 (70.18%)	82 (71.93%)	
>65	41 (25.31%)	52 (36.11%)		34 (29.82%)	32 (28.07%)	
BMI			0.925			0.472
<18.5	19 (11.73%)	19 (13.19%)		12 (10.53%)	17 (14.91%)	
18.5-23.9	116 (71.60%)	101 (70.14%)		85 (74.56%)	77 (67.54%)	
≥24	27 (16.67%)	24 (16.67%)		17 (14.91%)	20 (17.54%)	
Smoking history			0.912			1.000
no	64 (39.51%)	56 (38.89%)		42 (36.84%)	42 (36.84%)	
yes	98 (60.49%)	88 (61.11%)		72 (63.16%)	72 (63.16%)	
Drinking history			0.934			0.689
no	96 (59.26%)	86 (59.72%)		62 (54.39%)	65 (57.02%)	
yes	66 (40.74%)	58 (40.28%)		52 (45.61%)	49 (42.98%)	
Tumor location			0.524			0.963
upper	14 (8.64%)	12 (8.33%)		7 (6.14%)	8 (7.02%)	
middle	95 (58.64%)	76 (52.78%)		64 (56.14%)	63 (55.26%)	
lower	53 (32.72%)	56 (38.89%)		43 (37.72%)	43 (37.72%)	
Clinical stage			0.763			0.831
II	40 (24.69%)	35 (24.31%)		26 (22.81%)	29 (25.44%)	
III	105 (64.81%)	90 (62.50%)		77 (67.54%)	76 (66.67%)	
IVa	17 (10.49%)	19 (13.19%)		11 (9.65%)	9 (7.89%)	

MPR, major pathological response.

Survival analysis also revealed that the recurrence-free survival of patients achieving pCR were comparable to those of patients achieving MPR (P = 0.319 in the unmatched cohort; P = 0.456 in the matched cohort), as illustrated in **Figures 2C, D**.

### 3.4 The sensitivity analysis between non-pCR and pCR patients in MPR patients cohort

**Figure 3:**
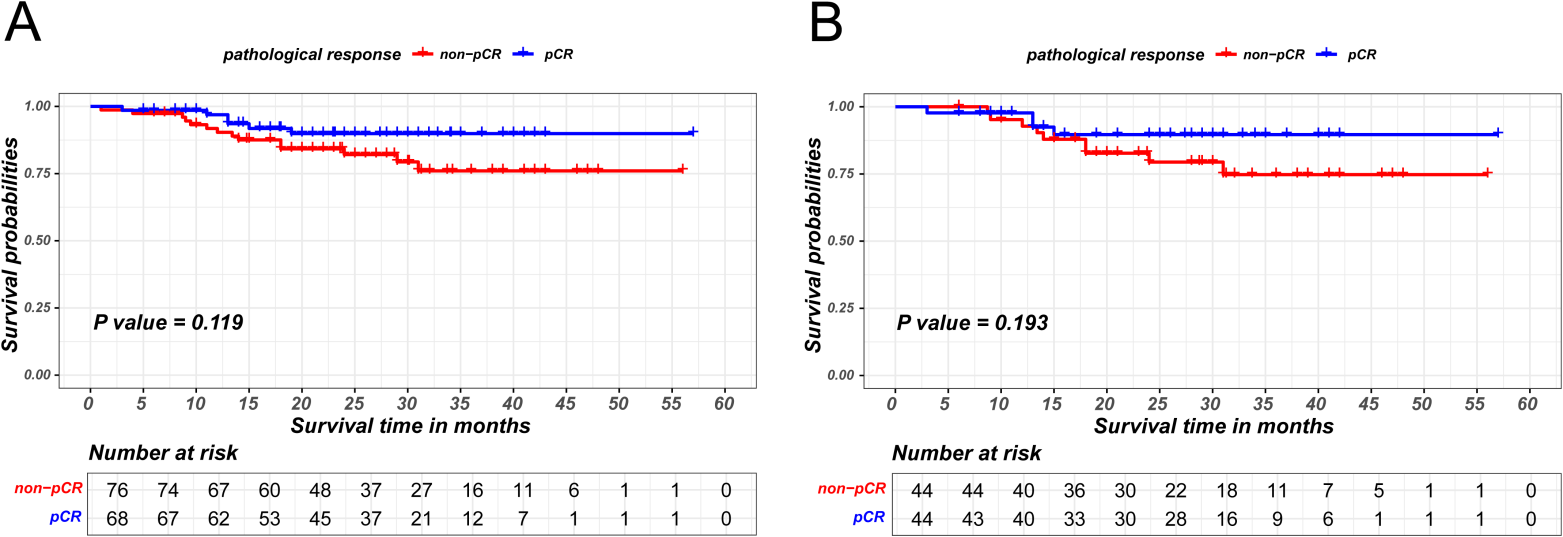
Kaplan-Meier survival curves of recurrence-free survival between pCR and non-pCR patients in MPR group before PSM **(A)** and after PSM **(B)**.

Given that patients achieving pCR are encompassed within the MPR cohort, a sensitivity analysis was utilized to explore survival difference between non-pCR and pCR patients within the MPR group. MPR patients were stratified in two groups based on pCR status and PSM was used to eliminate intergroup bias, as is shown in **Supplementary Table S2**. In the unmatched group, the Kaplan-Meier survival analysis indicated the absence of a statistically significant difference in disease-free survival between non-pCR and pCR patients within MPR cohort (P = 0.119). Similarly, the survival difference was not observed between two groups in the matched group (P = 0.193), as is shown in **Figure 3**.

### 3.5 The survival analysis of adjuvant therapy in MPR patients after nICT

**Figure 4:**
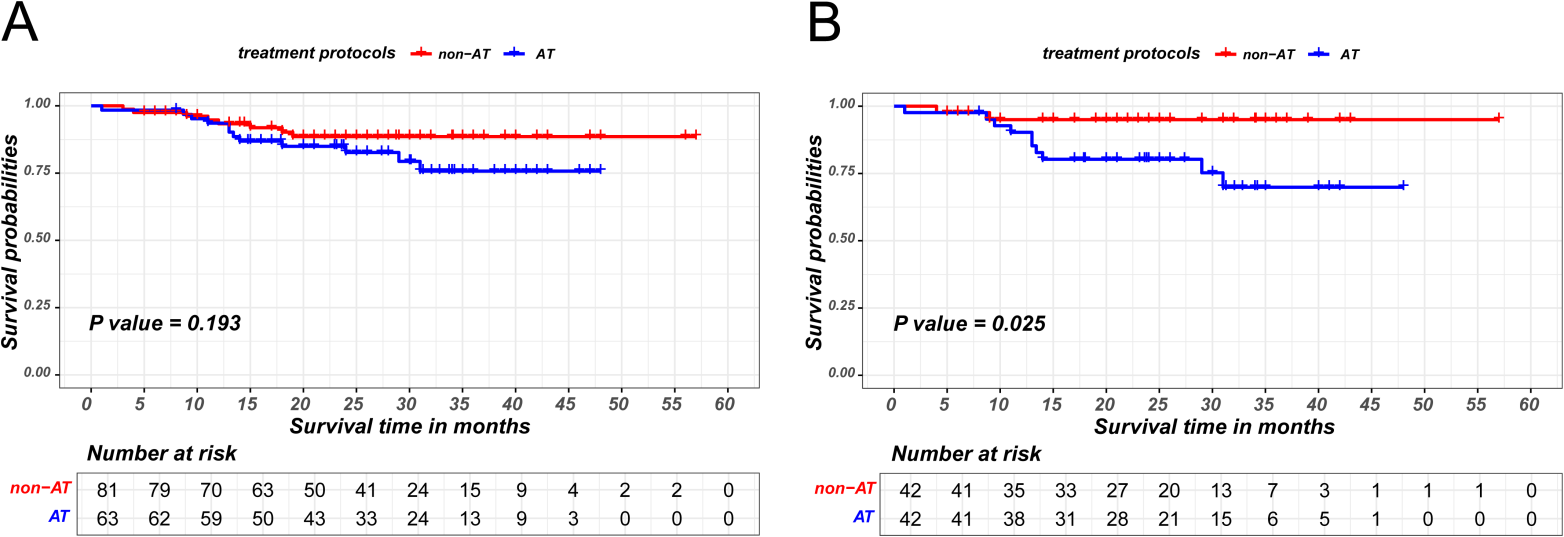
Kaplan-Meier survival curves of recurrence-free survival between non-AT and AT patients in MPR group before PSM **(A)** and after PSM **(B)**.

The prognostic significance of AT in ESCC patients achieving MPR was also investigated. Similarly, the MPR patients cohort was stratified into AT and non-AT group based on the administration of AT and employed PSM to minimize bias between the two groups, as detailed in **Supplementary Table S3**. The matching parameters of this propensity score matching included sex, age, smoking history, drinking history, BMI, tumor location, ypT stage and ypN stage. The Kaplan-Meier survival analysis revealed that AT did not confer an additional recurrence-free survival benefit for patients attaining MPR (P=0.193 in the unmatched cohort;P=0.025 in the matched cohort), as is shown in **Figure 4**.

### 3.6 Recurrence patterns comparison between MPR patients and pCR patients

**Figure 5:**
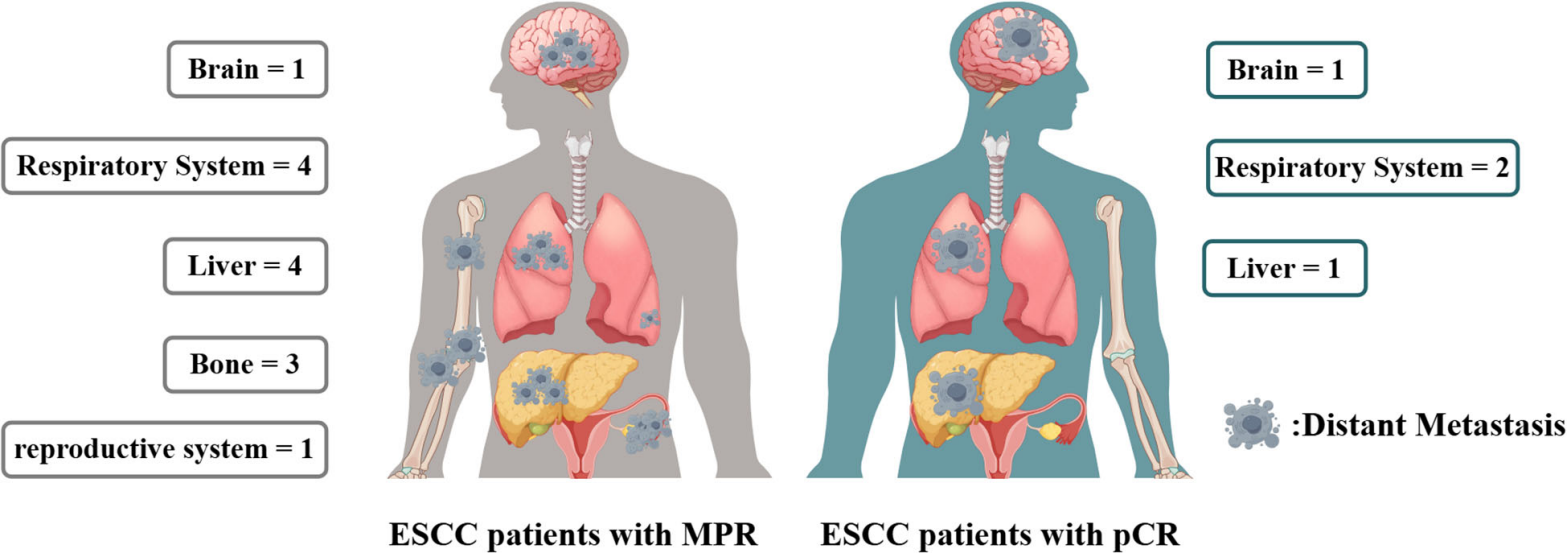
Distant metastasis site of ESCC patients of MPR and pCR.

In the cohort of patients with MPR, 20 individuals experienced recurrence, with 8 presenting solely with locoregional recurrence and another 8 exhibiting only distant metastasis. In contrast, among the pCR patients, 6 individuals experienced recurrence, of whom 2 had only locoregional recurrence and 3 had only distant metastasis. A correlation analysis indicated that the recurrence patterns between the two groups were comparable, as detailed in **Table 4** and **Figure 5**.

**Table 4:** Recurrence pattern in ESCC patients of MPR and pCR.

Variables	MPR patients	pCR patients	P value
Survival status			0.294
Recurrence-free	124 (86.11%)	62 (91.18%)	
Recurrence	20 (13.89%)	6 (8.82%)	
Recurrence pattern			1.000
Locoregional recurrence only	8 (40.00%)	2 (33.33%)	
Distant metastasis only	8 (40.00%)	3 (50.00%)	
Locoregional recurrence with distant metastasis	4 (20.00%)	1 (16.67%)	

## 4 Discussion

pCR and MPR are utilized as the principal endpoints in assessing the short-term efficacy of neoadjuvant trials. Previous researches showed that nICT could lead to higher rates of MPR and pCR compared to nCT alone (18, 19). Furthermore, in comparison to achieving a pCR, attaining a MPR is more readily achievable in ESCC patients undergoing neoadjuvant therapy (20). However, the question of whether ESCC patients achieving MPR have prognostic outcomes equivalent to those attaining pCR remains unclear. Our study found that patients who achieved MPR following nICT exhibited a more favorable prognosis compared to those who did not achieve MPR. Furthermore, their prognostic outcomes were comparable to those of patients who attained pCR.

This study demonstrated that patients exhibiting a MPR in the nICT group had a significantly improved prognosis compared to those without MPR, both prior to and following propensity score matching. The Cox regression analysis also identified MPR as an independent risk factor. From the perspective of tumor load, the status of MPR indicated that neoadjuvant therapy was particularly effective in reducing preoperative tumor burden and substantially decreasing the risk of tumor recurrence through the more efficient eradication of micro-metastases (21, 22). Furthermore, a distinctive characteristic of immunotherapy is its “tail effect” (23, 24). The tail effect is characterized by the sustained maintenance of therapeutic effects following treatment discontinuation, thereby providing long-term immune responses and better survival benefits for patients with advanced tumors. The development of the tail effect is linked to the “memory function” of immune cells, notably T cells. While, this effect requires a strong interaction between the patient’s immune system and the immunotherapeutic agent. Patients who achieve a MPR are more likely to exhibit an enhanced tail effect in the immune system during neoadjuvant therapy, which sustains anti-tumor activity postoperatively and results in improved clinical outcomes.

In our study, it was observed that the RFS of patients achieving a MPR was comparable to that of patients exhibiting a pCR. Sensitivity analysis indicated that, within the MPR cohort, the RFS of patients attaining pCR did not significantly differ from those who did not, corroborating previous findings (25). These findings implied that nICT may have yielded equivalent therapeutic outcomes to MPR patients and pCR patients. This observation also suggested that the MPR may serve as a surrogate endpoint for pCR in assessing the efficacy of nICT. Given the comparable RFS in MPR and pCR patients, MPR has the potential to merge as a more accessible and cost-effective prognostic indicator in clinical practice. The CROSS study demonstrated that nCRT significantly improves overall survival in EC patients compared to surgery alone; however, it does not confer enhanced survival benefits with respect to distant metastasis (26). Compared to nCRT, nICT offers a systemic therapeutic approach with a distinct advantage in the management of distant metastases, particularly in the control of microscopic metastasis (27).The integration of nICT and surgical intervention constitutes a comprehensive treatment modality that effectively manages locoregional recurrence and distant metastasis of tumors: nICT significantly reduces the quantity of tumor cells within the primary tumor and lymph nodes, especially in patients exhibiting drug sensitivity. During subsequent radical esophagectomy, the residual tumor cells can be effectively excised. The primary aims of neoadjuvant therapy are twofold: first, to achieve tumor downstaging, thereby enhancing the feasibility of curative surgical procedures (28); and second, to mitigate distant metastasis and micrometastasis of tumor cells through systemic drug administration, particularly when the tumor burden is substantial and the likelihood of metastasis is elevated. Numerous studies have documented that patients undergoing neoadjuvant therapy demonstrate superior recurrence-free survival rates in comparison to those receiving adjuvant therapy post-surgery, which implied that chemotherapy or immunotherapy might be more efficacious in managing distant metastasis when the tumor burden is elevated, as is the case prior to surgical intervention (29, 30). Both MPR and pCR reflect a significant sensitivity to the treatment protocol, showing the effective eradication of a considerable proportion of cancer cells. As a result, the prognosis for patients with MPR is notably enhanced due to the effective local tumor control attained through following standardized esophagectomy. This improvement may account for the similar RFS observed between ESCC patients with MPR and those with pCR following surgery.

In our study, pCR was found to be a prognostic factor but not an independent predictor of survival outcome, consistent with findings reported in other studies (31–33). This indicated that pCR can be a useful short-term indicator for evaluating treatment efficacy, but it may not be a reliable surrogate for long-term outcomes, especially in ESCC patients after nICT. The CheckMate 577 trial has shown that adjuvant immunotherapy can enhance survival outcomes for EC patients who have residual tumor cells after nCRT (34). Nonetheless, there are currently no established guidelines delineating the indications for adjuvant therapy in patients undergoing nICT. In our prior investigations, we found that the prognosis for ESCC patients who attained a pCR following nICT was unaffected by adjuvant therapy (35). In this study, it was observed that adjuvant therapy did not provide additional advantages in terms of disease-free survival for patients achieving a MPR, aligning with the outcomes observed in patients with pCR. Prior study has indicated that compared to those who did not undergo AT, the receipt of AT showed shorter disease-free survival following neoadjuvant chemoradiotherapy and surgery (36). Similarly, Yan’s study showed that adjuvant therapy failed to improve survival outcomes in patients receiving neoadjuvant chemotherapy and instead indicated a trend toward worse prognosis (37). It is well recognized that AT does not benefit all ESCC patients. There are three possible reasons for negative impact of AT. Firstly, postoperative impairments such as reduced food intake and swallowing difficulties and postoperative complications can significantly weaken the immune system of ESCC patients (38–40). In such circumstances, adjuvant therapy may further weaken the immune system’s ability to target cancer cells, potentially leading to disease recurrence (41, 42). Secondly, damage to the integrity of Lymph node, particularly as a result of systematic lymphadenectomy, may impair the activation of anti-tumor T cells, consequently diminishing the effectiveness of subsequent immunotherapeutic interventions (43, 44). Thirdly, the reduction of tumor burden after surgery may weaken the efficacy of adjuvant therapy. These findings suggested that clinicians might consider implementing a surveillance approach for patients achieving MPR after surgery, akin to the strategy employed for those with pCR. Besides, clinicians should take both MPR and pCR into account when assessing the prognosis of LA-ESCC patients, as this may facilitate the development of more tailored treatment strategies. However, we still believe this finding still requires further validation from through prospective, nationwide, multi-center clinical trials.

In the nICT cohort of patients with ESCC, the recurrence patterns observed in patients achieving MPR were analogous to those in patients achieving pCR, encompassing both distant metastasis and locoregional recurrence. Furthermore, in instances of distant metastasis, the predominant sites for both MPR and pCR patients were the brain, lungs, and liver. The analogous recurrence patterns and distribution of metastasis sites indicated the necessity for equal consideration to two patients group during follow-up. The recurrence pattern similarities between MPR and pCR patients also implied that these groups could be perceived as having comparable risk levels when devising follow-up strategies. Nonetheless, it is imperative to acknowledge that despite attaining MPR or pCR, patients remain at risk for recurrence. Ongoing surveillance is still essential to enhance prognostic outcomes.

**Figure 6:**
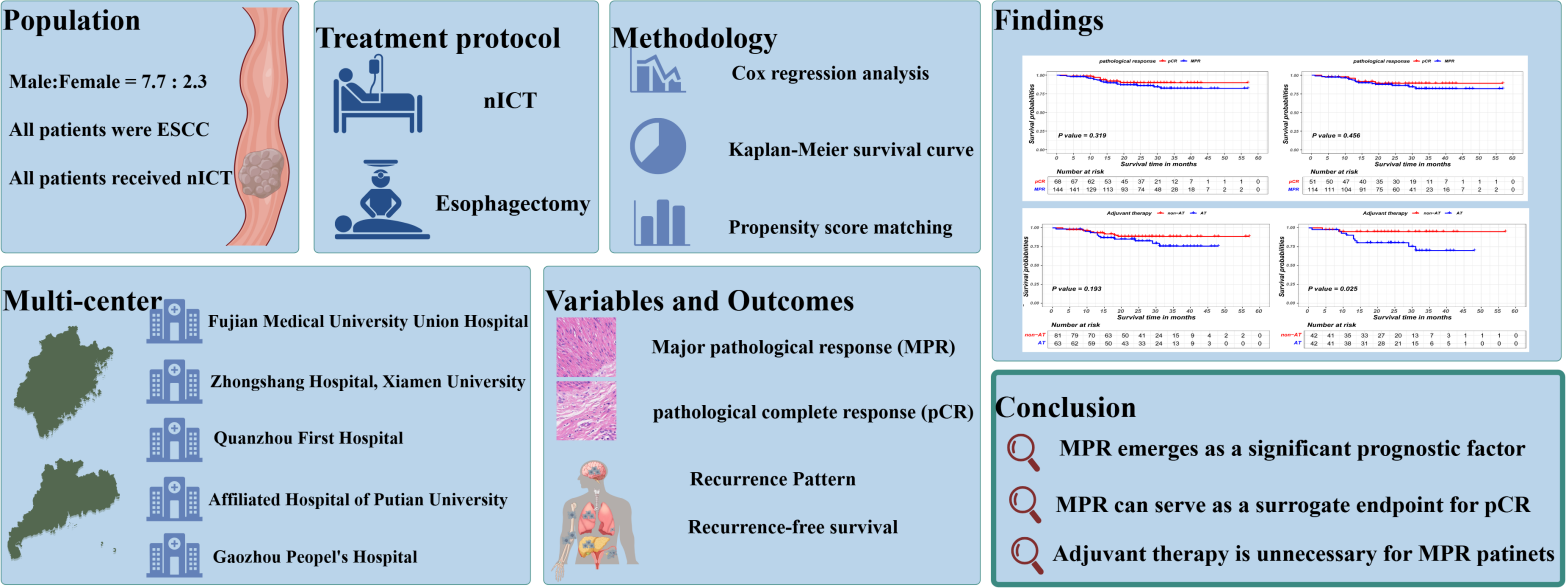
Visual abstract of this study.

This study has several limitations. First, despite being a multi-center study and employing rigorous selection criteria to mitigate selection bias, its retrospective design introduces inherent limitations. Secondly, this study was limited to patients with esophageal squamous cell carcinoma, and the extent to which our findings can be generalized to patients with adenocarcinoma remains uncertain. In future studies, we plan to conduct a separate analysis of esophageal adenocarcinoma. This approach will be pursued in subsequent research, although it will require additional time to collaborate with more centers due to the relatively low incidence of esophageal adenocarcinoma in China. The visual abstract of this study is shown in **Figure 6**.

## 5 Conclusion

In conclusion, MPR emerges as a significant prognostic factor in ESCC patients undergoing nICT, with outcomes similar to those achieving pCR. These findings warrant consideration of MPR as a potential surrogate endpoint for pCR and highlight the need to refine treatment strategies based on pathological responses. Further studies are necessary to confirm these results and to explore the integration of MPR into clinical practice, potentially leading to more personalized and effective treatment approaches for ESCC patients.

## Data Availability

The raw data supporting the conclusions of this article will be made available by the authors, without undue reservation.
