# Supplementary material for: The prognostic power of major pathological response in esophageal squamous cell carcinoma patients undergoing neoadjuvant chemoimmunotherapy: a multi-center cohort study
Source: Front Immunol. 2025 Jul 7;16:1599526. doi: 10.3389/fimmu.2025.1599526 (PMC12277351; doi:10.3389/fimmu.2025.1599526)
Supplement: Supplementary file 1 [file DataSheet1.docx]

Tabel S1. The detail information of drugs used in nICT and AT

| Variables | N | （%） |
| --- | --- | --- |
| **Neoadjuvant chemotherapy regimen** |  |  |
| docetaxel/paclitaxel with platinum | 304 | 99.35% |
| Others* | 2 | 0.65% |
| **Immune drugs** |  |  |
| sintilimab | 71 | 23.20% |
| camrelizumab | 131 | 42.81% |
| tislelizumab | 37 | 12.09% |
| toripalimab | 16 | 5.23% |
| pembrolizumab | 51 | 16.67% |
| **Adjuvant therapy regimen** |  |  |
| aICT | 98 | 64.05% |
| aCT | 39 | 25.49% |
| aIT | 16 | 10.46% |

aICT：adjuvant chemoimmunotherapy; aCT: adjuvant chemotherapy; aIT: adjuvant immunotherapy.

*: 2 patients take platinum with tegafur as neoadjuvant chemotherapy regimen.

**Table S2**: Characteristics comparison of pCR patients and non-pCR patients in the MPR group before and after matching.

| Variables | Before PSM | | | After PSM | | |
| --- | --- | --- | --- | --- | --- | --- |
|  | non-pCR | pCR | P value | non-pCR | pCR | P value |
| **Sex** |  |  | 0.552 |  |  | 0.803 |
| male | 57 (75.00%) | 48 (70.59%) |  | 33 (75.00%) | 34 (77.27%) |  |
| female | 19 (25.00%) | 20 (29.41%) |  | 11 (25.00%) | 10 (22.73%) |  |
| **Age** |  |  | 0.877 |  |  | 0.467 |
| ≤65 | 49 (64.47%) | 43 (63.24%) |  | 31 (70.45%) | 34 (77.27%) |  |
| ＞65 | 27 (35.53%) | 25 (36.76%) |  | 13 (29.55%) | 10 (22.73%) |  |
| **BMI** |  |  | 0.770 |  |  | 1.000 |
| ＜18.5 | 9 (11.84%) | 10 (14.71%) |  | 3 (6.82%) | 4 (9.09%) |  |
| 18.5-23.9 | 53 (69.74%) | 48 (70.59%) |  | 35 (79.55%) | 34 (77.27%) |  |
| ≥24 | 14 (18.42%) | 10 (14.71%) |  | 6 (13.64%) | 6 (13.64%) |  |
| **Smoking history** |  |  | 0.621 |  |  | 0.829 |
| no | 31 (40.79%) | 25 (36.76%) |  | 19 (43.18%) | 18 (40.91%) |  |
| yes | 45 (59.21%) | 43 (63.24%) |  | 25 (56.82%) | 26 (59.09%) |  |
| **Drinking history** |  |  | 0.374 |  |  | 1.000 |
| no | 48 (63.16%) | 38 (55.88%) |  | 26 (59.09%) | 26 (59.09%) |  |
| yes | 28 (36.84%) | 30 (44.12%) |  | 18 (40.91%) | 18 (40.91%) |  |
| **Tumor location** |  |  | 0.432 |  |  | 0.424 |
| upper | 5 (6.58%) | 7 (10.29%) |  | 4 (9.09%) | 1 (2.27%) |  |
| middle | 38 (50.00%) | 38 (55.88%) |  | 24 (54.55%) | 24 (54.55%) |  |
| lower | 33 (43.42%) | 23 (33.82%) |  | 16 (36.36%) | 19 (43.18%) |  |
| **Clinical stage** |  |  | 0.465 |  |  | 0.457 |
| II | 16 (21.05%) | 19 (27.94%) |  | 8 (18.18%) | 13 (29.55%) |  |
| III | 48 (63.16%) | 42 (61.76%) |  | 30 (68.18%) | 26 (59.09%) |  |
| IVa | 12 (15.79%) | 7 (10.29%) |  | 6 (13.64%) | 5 (11.36%) |  |

MPR:major pathological response;

**Table S3** Characteristics comparison of AT patients and non-AT patients in the MPR patients group before and after matching.

| Variables | Before PSM | | | After PSM | | |
| --- | --- | --- | --- | --- | --- | --- |
|  | non-AT | AT | P value | non-AT | AT | P value |
| **Sex** |  |  | 0.125 |  |  | 0.794 |
| male | 55 (67.90%) | 50 (79.37%) |  | 33 (78.57%) | 32 (76.19%) |  |
| female | 26 (32.10%) | 13 (20.63%) |  | 9 (21.43%) | 10 (23.81%) |  |
| **Age** |  |  | 0.018 |  |  | 0.811 |
| ≤65 | 45 (55.56%) | 47 (74.60%) |  | 29 (69.05%) | 30 (71.43%) |  |
| ＞65 | 36 (44.44%) | 16 (25.40%) |  | 13 (30.95%) | 12 (28.57%) |  |
| **BMI** |  |  | 0.956 |  |  | 0.948 |
| ＜18.5 | 11 (13.58%) | 8 (12.70%) |  | 5 (11.90%) | 5 (11.90%) |  |
| 18.5-23.9 | 56 (69.14%) | 45 (71.43%) |  | 32 (76.19%) | 31 (73.81%) |  |
| ≥24 | 14 (17.28%) | 10 (15.87%) |  | 5 (11.90%) | 6 (14.29%) |  |
| **Smoking history** |  |  | 0.389 |  |  | 0.821 |
| no | 29 (35.80%) | 27 (42.86%) |  | 15 (35.71%) | 16 (38.10%) |  |
| yes | 52 (64.20%) | 36 (57.14%) |  | 27 (64.29%) | 26 (61.90%) |  |
| **Drinking history** |  |  | 0.134 |  |  | 0.825 |
| no | 44 (54.32%) | 42 (66.67%) |  | 24 (57.14%) | 25 (59.52%) |  |
| yes | 37 (45.68%) | 21 (33.33%) |  | 18 (42.86%) | 17 (40.48%) |  |
| **Tumor location** |  |  | 0.183 |  |  | 1.000 |
| upper | 5 (6.17%) | 7 (11.11%) |  | 3 (7.14%) | 3 (7.14%) |  |
| middle | 48 (59.26%) | 28 (44.44%) |  | 19 (45.24%) | 20 (47.62%) |  |
| lower | 28 (34.57%) | 28 (44.44%) |  | 20 (47.62%) | 19 (45.24%) |  |
| **ypT stage** |  |  | 0.26 |  |  | 1.000 |
| T_0-2_ | 70 (86.42%) | 50 (79.37%) |  | 37 (88.10%) | 37 (88.10%) |  |
| T_3-4_ | 11 (13.58%) | 13 (20.63%) |  | 5 (11.90%) | 5 (11.90%) |  |
| **ypN stage** |  |  | 0.254 |  |  | 0.608 |
| N_0_ | 61 (75.31%) | 42 (66.67%) |  | 31 (73.81%) | 33 (78.57%) |  |
| N_1-3_ | 20 (24.69%) | 21 (33.33%) |  | 11 (26.19%) | 9 (21.43%) |  |
